# Silencing of carbonic anhydrase I enhances the malignant potential of exosomes secreted by prostatic tumour cells

**DOI:** 10.1111/jcmm.14265

**Published:** 2019-03-27

**Authors:** Radivojka Bánová Vulić, Martina Zdurienčíková, Silvia Tyčiaková, Oldřich Benada, Mária Dubrovčáková, Ján Lakota, Ľudovít Škultéty

**Affiliations:** ^1^ Biomedical Research Center SAS Bratislava Slovak Republic; ^2^ Institute of Microbiology of the CAS, v.v.i. Prague Czech Republic; ^3^ St. Elizabeth Cancer Institute Bratislava Slovak Republic; ^4^ Center of Experimental Medicine SAS Bratislava Slovak Republic

**Keywords:** carbonic anhydrase I, exosomes, LC‐MS, malignant potential, PC3 cells, siCA1, siMock

## Abstract

We report results showing that the silencing of carbonic anhydrase I (siCA1) in prostatic (PC3) tumour cells has a significant impact on exosome formation. An increased diameter, concentration and diversity of the produced exosomes were noticed as a consequence of this knock‐down. The protein composition of the exosomes' cargo was also altered. Liquid chromatography and mass spectrometry analyses identified 42 proteins significantly altered in PC3 siCA1 exosomes compared with controls. The affected proteins are mainly involved in metabolic processes, biogenesis, cell component organization and defense/immunity. Interestingly, almost all of them have been described as ‘enhancers' of tumour development through the promotion of cell proliferation, migration and invasion. Thus, our results indicate that the reduced expression of the CA1 protein enhances the malignant potential of PC3 cells.

## 1 INTRODUCTION

Exosomes are saucer‐shaped microvesicles, 30‐180 nm in diameter, enveloped in a lipid bilayer. They contain various molecular constituents of their cell of origin, such as proteins, DNA, mRNA, as well as non‐coding RNAs. It has been suggested that they play an influential role in cell‐to‐cell signalling, the exchange of genetic information and the reprogramming of the recipient cells.^1^, ^2^ Thus, exosomes derived from tumour cells may trigger tumour initiation, angiogenesis, growth, the progression of the disease, or the formation of metastases. They may also play an important role in tumour‐stroma interactions, chemotherapy and drug resistance.^4^, ^5^ These vesicles carry messages from tumour cells to immune or stromal cells which may result in the prevention of immune recognition and modification of their microenvironment. Tumour cells release an enormous amount of exosomes. Compared with healthy controls, their numbers are increased in the plasma of some cancer patients. A clear correlation between the total amount of tumour exosomes and the stage of tumour development was also reported in ovarian and prostate cancer patients.^7^ In this respect, some exosomal proteins and miRNAs have been suggested as diagnostic and prognostic indicators for lung cancer, esophageal squamous cell carcinoma, prostate cancer, breast cancer, glioblastoma, ovarian cancer and other cancer types.^8^, ^9^ Interestingly, the increased abundance of some specific exosomal miRNAs and proteins has been positively correlated with the stage and degree of prostate cancer progression.^10^, ^11^ Since exosomes reflect the pathological state of the secretory cells, they have become attractive new biomarkers for the diagnosis and prognosis of cancer.^1^, ^10^ Furthermore, they represent a novel therapeutic strategy for the treatment of cancers.^1^, ^10^

Besides being ubiquitous in all life forms, it was found that carbonic anhydrases perform numerous activities in a variety of organisms. Primarily they catalyse the hydration of carbon dioxide to a bicarbonate ion and a proton.^12^ This reaction is reversible and the metalloenzymes can accomplish it in both directions, forward and reverse.^13^ This interconversion is essential for many biological processes, which require acid‐base balance and depend on spatially and temporally regulated ion transport in various subcellular compartments and across the plasma membrane. Abnormal levels or activities of these enzymes have been associated with many disorders such as obesity, gastric ulcers, glaucoma, acid‐base imbalances, cancer and epilepsy.^14^ Carbonic anhydrase I (CA I) is a zinc metalloenzyme belonging to the α CA family.^12^ It is involved in pH homeostasis, respiration, erythroid differentiation and some pathological processes such as anaemia, chronical acidosis, proliferative diabetic retinopathy and diabetic macular and vasogenic oedemas.^14^, ^15^ Variations in the expression of CA I have recently been associated with some malignancies. A low level of CA I in colonic epithelial cells was found to be a specific marker for the prediction of colorectal cancer.^18^ On the other hand, CA I was highly expressed in the sera of patients with stage I non‐small cell lung cancer (NSCLC).^19^ Even the plasma of patients with prostate cancer contains an increased level of CA I compared with healthy controls.^20^ Thus, the elevated level of the CA I protein in the plasma or serum may represent a promising biomarker for both prostate cancer and early stage NSCLC. Interestingly, the autoantibodies against CA I were also linked to the progress of malignant diseases. Lakota et al have reported a significant increase in these autoantibodies in the sera of patients with tumours that spontaneously regressed after high‐dose therapy and autologous stem cell transplantation.^21^ Thus, the presence of autoantibodies against CA I in patients' sera could be put forward as a marker of a good prognosis. Further in vitro research on tumour cells showed that treatment with these sera may result in the up‐regulation of the CA1 mRNA expression, which can be linked to the down‐regulation of the mRNAs encoding structural proteins of basal lamina, the cytoskeleton, WNT7B and collagen triple helix repeat containing 1 (CTHRC1).^22^ On the other hand, CA1 mRNA silencing via the RNA interference system in PC3 tumour cells enhanced the expression of some of the extracellular matrix (ECM) proteins.^23^

To reveal the role of CA I in prostatic cancer development, a more thorough study is required. The mRNA CA1 was silenced in PC3 cells and the secreted exosomes were subsequently isolated and characterized using different methods. A comprehensive proteomic analysis was then performed using mass spectrometry to identify the differences in the protein composition of the exosomal cargo of these cells developed due to the changes in mRNA CA1 expression.

Taken together, the comprehensive characterization of exosomes derived from PC3 prostate cells, which have different mRNA CA1 expression, shows that the knock‐down of CA1 mRNA in PC3 cells alters the exosomal pattern of cancer cells and enhances their malignant potential.

## 2 MATERIALS AND METHODS

### 2.1 Cell culture handling

A human prostate adenocarcinoma PC3 cell line derived from bone metastases (ATCC®‐CRL 1435TM) was grown in high‐glucose (4.5 mg/mL) Dulbecco's Modified Eagle's Medium (*Biochrom AG, Germany*), supplemented with 10% foetal calf serum (FCS) (*Lonza BioWhittaker,* *Switzerland*) and gentamicin (*Sandoz,* *Switzerland*) at 37°C in humidified air with 5% CO_2_.

### 2.2 Transient silencing of CA1 gene

For the transient silencing of the *CA1* gene, PC3 cells were seeded at a density of 1.5 × 10^6^ cells in T25 cell culture flasks. Transient CA1 knockdown cells were produced by transfection with SmartPool CA1 siRNA specific oligonucleotides (*Dharmacon, GE Healthcare, USA*) using DharmaFECT^™^ *(GE Healthcare, USA)* according to the manufacturer's recommendations, while siMock oligonucleotides were used as a control. Eighteen hours after transfection, the medium was changed to a (FCS)/antibiotic (ATB) free medium and the cells were incubated for 48 hours at 37°C. Conditioned media were collected for exosome isolation and the cells were lysed in a RIPA buffer for Western blot analysis.

### 2.3 Exosome preparation and purification

For the isolation of PC3 exosomes, PC3 silencing of carbonic anhydrase I (siCA1) as well as PC3 siMock (1.5 × 10^6^ each) cells were cultured in T25 cell culture flasks 50 mL (two flasks with 25 mL medium each) of FCS/ATB free medium. After 48 hours (max 80% confluency), the media were collected (100 mL from PC3 siCA1 and siMock cells), centrifuged (300g for 10 minutes) to remove cell debris and filtered through a 0.22 µm filter (*Merck Millipore,* *USA*). The pre‐cleared medium was concentrated to 2 mL using a 100 kDa MWCO Amicon Ultra Centrifugal Filter (*Merck Millipore, USA*). The concentrated media samples (2 mL) were centrifuged at 3 000 *g* for 15 minutes at 4°C, and subsequently, the exosomes were precipitated using a ExoQuick‐TC exosome precipitation solution *(System Biosciences, USA)*according to the manufacturer's instructions. The medium was then briefly mixed with 1/5 V (400 µL) of an ExoQuick‐TC exosome precipitation solution and refrigerated overnight (at least 12 hours) at 4°C. The next day, an ExoQuick‐TC/medium mixture was centrifuged at 1 500 *g* for 30 minutes. After centrifugation, the exosomes appeared as beige pellets and were washed once in a phosphate‐buffered saline (PBS) solution, and resuspended in an appropriate volume of PBS.

### 2.4 Nanoparticle tracking analysis

A NanoSight NS500 (*Malvern Instruments Ltd., Malvern, UK*) equipped with a sCMOS Trigger camera and a 405 nm laser was used to measure the concentration and size distribution of the exosomes isolated from PC3 siCA1 and PC3 siMock cells. The measured data were analysed using the noparticle tracking analysis (NTA) 2.3 analytical software. NTA is based on capturing the Brownian motion and light scattering properties to obtain particle size distributions and concentrations. Each sample was diluted in PBS prior to the measurements to optimize the number of particles (from 60x to 3600x dilution; used dilution:1800x). Samples were measured in triplicates in 60‐second videos with manual shutter and gain adjustments. All measurements were performed at room temperature.

### 2.5 Transmission electron microscopy

Five mocrolitres of PC3 siCA1 and siMock exosome samples were placed onto glow‐discharge activated carbon/formvar grids and were allowed to adsorb for 60 seconds at room temperature.^24^ After adsorption, the grids were negatively stained with 1% ammonium molybdate + 0.1% trehalose for 30 seconds.^25^ The grids were then air‐dried and examined in a FEI Morgagni *(FEI, Brno, Czech Republic)* transmission electron microscope at 80 kV. Digital images were recorded at magnifications of 18000x and 56000x with a MegaView III slow‐scan CCD camera (*formerly Olympus Soft Imaging Solutions, now EMSIS GmbH, Germany*) and processed with the AnalySis3.2 software suite (*formerly Olympus Soft Imaging Solutions, now EMSIS GmbH, Germany*) using embedded modules (Shading correction and “Optimize 16‐bit image for 8‐bit display”). No other image manipulation was used.

### 2.6 Gene expression analyses

For reverse transcriptase quantitative PCR (RT‐qPCR), the total RNA was extracted from PC3 siCA1 and PC3 siMock cells with a NucleoSpin RNA II kit (*Macherey‐Nagel, Dueren, Germany*). The RNA was depleted from genomic DNA using DNase treatment (DNase I, RNase‐free; *Thermo Fisher Scientific, Waltham, MA, USA*) and 1.15 µg of total RNA was reverse transcribed with a SensiFAST cDNA Synthesis kit (*Bioline, UK*). RT‐qPCR was performed in Brilliant III Ultra‐Fast SYBR QPCR Master Mix (*Agilent Technologies, USA*), 0.25 pmol/µL concentration of primers and 0.5 µL template cDNA in Bio‐Rad 96FX cycler *(Bio‐Rad, USA)* and analysed using the Bio‐Rad CFX Manager software 1.6 as normalized fold expression (2^−ΔΔCt^ method). Primer sequences for the CA1 gene and HPRT1 reference gene were as follows:

CA1sense 5′‐TAAAACCAAGGGCAAACGAG‐3′,

CA1antisense 5′‐GGCTGTGTTCTTGAGGAAGG‐3′,

HPRT1sense 5′‐TGACCAGTCAACAGGGGACA‐3′,

HPRT1antisense 5′‐ACTGCCTGACCAAGGAAAGC‐3′.

All oligonucleotides were synthesized by Metabion, Int. (*Martinsried, Germany*).

### 2.7 ELISA for exosomes detection

Nanoparticles (10^6^ nanoparticles measured by NanoSight NS500) were seeded in a volume of 50 µL per well in PBS in 96 well‐plates (*Greiner, Austria*) and incubated overnight at 37°C. After that, nanoparticles were washed five times with 0.05% Tween 20 in PBS (washing solution). A blocking solution (1% milk + 0.05% Tween 20 in PBS) was added at room temperature for 1 hour. For detection of TSG 101, exosomes were lysed with 0.1% Triton X‐100 in PBS for 3 minutes at room temperature and five times washed before adding the primary antibody. After five washings, the primary anti‐CD63 (rabbit) or anti‐TSG 101 (goat) polyclonal antibodies *(Santa Cruz Biotechnology, USA*) were diluted at a concentration of 2 µg/mL. Fifty microlitres of primary antibodies were added per well and incubated for 1 hour at 37°C. After five washings, the plate was incubated with adequate HRP‐conjugated secondary antibodies CD63 anti‐rabbit diluted at 1:500 (*Santa Cruz Biotechnology, USA*) and TSG 101 anti‐goat diluted at 1:1500 (*Santa Cruz Biotechnology, USA*) in a blocking solution for 1 hour at room temperature. After the final five washings, the reaction was developed with OPD for 30 minutes *(Merck, USA)*, the reaction was stopped with 2 mol/L H_2_SO_4_ and optical densities were recorded at 492 nm.

### 2.8 Immunoblotting

Isolated exosomes were mixed with a five times Laemmli Sample Buffer (10% β‐mercaptoethanol, *Bio‐Rad, USA*), diluted with PBS to acquire an equal concentration of protein for Western blotting, and boiled for 10 minutes at 95°C. Samples of 20 µg of exosomal proteins were run in 12% acrylamide SDS‐PAGE and stained in Coomassie blue. For Western blot analysis, samples separated in SDS‐PAGE were blotted onto the PVDF membrane (*Millipore, Germany*). The membrane was blocked for 2 hours in a blocking buffer containing 5% non‐fat milk in PBS with 0.1% Tween 20. Incubation with primary antibodies: CD9 rabbit antibody diluted at 1:1000 (*Cell Signaling, USA*) and TSG 101 was performed overnight at 4°C. For CA I detection, azide‐free CA I mouse monoclonal antibody (*Moravian Biotechnology, Czech Republic*), was diluted at 1:1000 in 3% BSA in TBS‐T. Then the membrane was washed with 0.1% Tween 20 in PBS (3 × 10 minutes), incubated for 1 hour (RT) with secondary antibody (*Sigma‐Aldrich, USA*), washed again (3 × 10 minutes) and developed with the enhanced chemiluminescence detection system.

### 2.9 Sample preparation for MS analysis

Exosomes were isolated from three biological replicates of mRNA CA1 knockdown and control cells. The amount of nanoparticles was determined using a NanoSight NS500, and exosomal protein concentration was measured using BCA. Exosomes were sonicated (water bath, 3 minutes) and diluted 1:1 with 100 mmol/L ammonium bicarbonate. Subsequently, exosomal proteins were reduced with 10 mmol/L dithiothreitol (DTT, *Sigma‐Aldrich, USA*) in 100 mmol/L ammonium bicarbonate buffer (45 minutes, 56°C) and S‐alkylated with 50 mmol/L iodoacetamide (IAA, *Sigma‐Aldrich, USA*) in 100 mmol/L ammonium bicarbonate buffer (30 minutes, RT). After neutralization of IAA, the proteins were digested with trypsin (*(Promega, USA)*enzyme: substrate ratio: 1:50 in 50 mmol/L ammonium bicarbonate buffer) overnight at 37°C under gentle shaking. Enzymatic cleavage was stopped with the addition of 5% formic acid (FA, *Sigma Aldrich, USA*). Subsequently, each sample was desalted using a MicroTrap Peptide 6 PK cartridge *(Bruker, Germany)*. The extracts were concentrated in the SpeedVac (*Eppendorf, Germany*) to 0.5 μg/μL.

### 2.10 LC‐MS analysis

Aliquots of purified complex peptide mixtures (1 μg) were separated in technical triplicate, using nanoAcquity UHPLC (*Waters*). Samples were loaded onto a Symmetry C18 trap column (20 mm length, 180 μm diameter, 5 μm particles size). After 3 minutes of desalting/concentration by 1%, acetonitrile containing 0.1% formic acid at a flow rate 10 μL/min, peptides were introduced to a BEH130 C18 analytical column (200 mm length, 75 μm diameter, 1.7 μm particle size). For the thorough separation, a 60‐min gradient of 5%‐40% acetonitrile with 0.1% formic acid was applied at a flow rate of 300 nL/min. The column outlet was connected to a PicoTip emitter (360 μm outer diameter, 20 μm inner diameter, 10 μm tip diameter) and samples were nanosprayed (3.4 kV capillary voltage) to the quadrupole time‐of‐flight mass spectrometer Q‐TOF Premier (*Waters*).

Spectra were recorded in a data‐independent manner in MSE mode. This mode uses alternate scans at low (4 eV) and high (20‐40 eV ramp) collision energies to obtain full‐scan mass data for both precursors and fragments in a single run. Ions with 50‐1950 m/z were detected in both channels. The spectral acquisition scan rate was 1.2 seconds, with a 0.05 seconds inter‐scan delay. The external mass calibrant Glu1‐Fibrinopeptide B (500 fmol/mL) was infused through the reference line at a flow rate of 500 nL/min and employed for mass correction.

### 2.11 Relative label‐free quantification

Data processing was done in Progenesis QI (*Waters*) v. 4.0. For peak identification the following threshold parameters were applied: low energy: 140 counts and high energy: 30 counts. Precursors and fragment ions were paired using correlations with chromatographic elution profiles in low/high energy traces. Then, the peaks' retention times were aligned across all chromatograms. Peak intensities were normalized to all ions, assuming the majority of signals are unaffected by experimental conditions. The label‐free quantification relied on measurement of peak areas of precursor peptides. For the protein identification, the Ion Accounting v. 4.0 (*Waters*) search algorithm was applied. The reference sequence file downloaded from https://www.uniprot.org/proteomes/UP000005640 contained 73,101 protein sequences. The workflow parameters for the protein identification searches were as follows: one possible missed cleavage utilizing trypsin as the protease, a fixed modification of Cys (carbamidomethylation), possible modifications of Met (oxidation) and Asn/Gln (deamidation). The precursor and peptide fragment mass tolerances were automatically determined by the software. Protein identification was limited to less than 4% false discovery rate against the randomized database, which was applied at the individual peptide level. Identifications were accepted if at least two distinct reliable peptides (score ≥ 6, mass accuracy < 25 ppm) matched the protein sequence. Protein grouping feature was then applied to show only hits with unique peptides. Their ratios were then used for relative quantification. We considered only those proteins as differentially abundant which reached at least 1.5‐fold change and their ANOVA *P*‐values were lower than 0.05. The values have been presented as means ± standard deviations of three biologicals and two technical replicates. The mass spectrometry proteomics data have been deposited to the ProteomeXchange Consortium via PRIDE^26^ partner repository with dataset identifier PXD 313942.

## 3 RESULTS

### 3.1 Efficacy of the CA1 silencing

**Figure 1:**
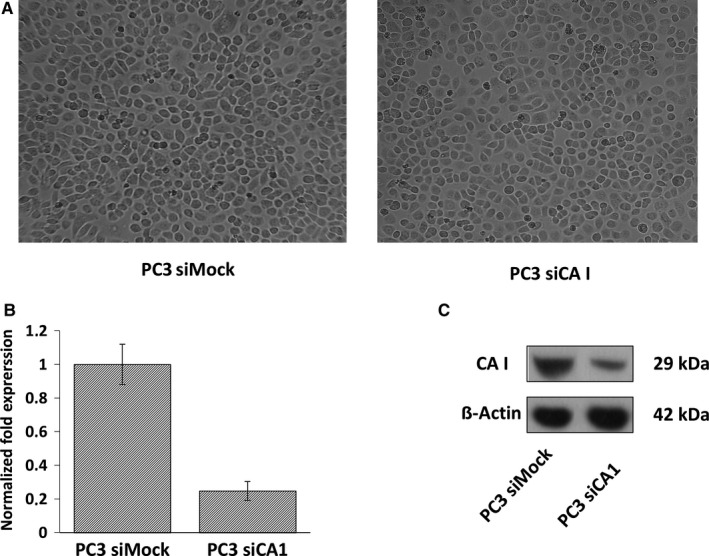
Efficacy of CA1 transcriptional silencing. (A) A light‐microscope image of PC3 cells after silencing of carbonic anhydrase I (siCA1) transfection compared to PC3 cells transfected with Mock siRNA. Eighteen hours after transfection, the medium was changed to a foetal calf serum (FCS)/antibiotic (ATB) free medium and the cells were incubated for 48 h at 37°C. Magnification 100×. (B) Gene expression analysis of silenced CA1 mRNA; reverse transcriptase quantitative PCR (RT‐qPCR) analysis. (C) Western blot analysis indicating reduced amount of Carbonic anhydrase I (CA I) protein after CA1 mRNA silencing in PC3 tumour cells. Beta‐actin was used as a normalization control

The knockdown of the CA1 gene was performed using the siRNA—SMARTpool system targeting the CA1 mRNA. The CA1 siRNA—transfected PC3 cells did not show any differences in morphology (Figure 1A) compared to the negative control (PC3 siMock). The silencing efficiency of the CA1 mRNA was confirmed by qRT‐PCR and Western blot analysis. The result showed a 75% reduction in mRNA expression in the PC3 siCA1‐transfected cells (Figure 1B). Accordingly, the abundance of CA I protein was also decreased (Figure 1C).

### 3.2 Characterization of exosomes derived from PC3 siCA1 and PC3 siMock cells

**Figure 2:**
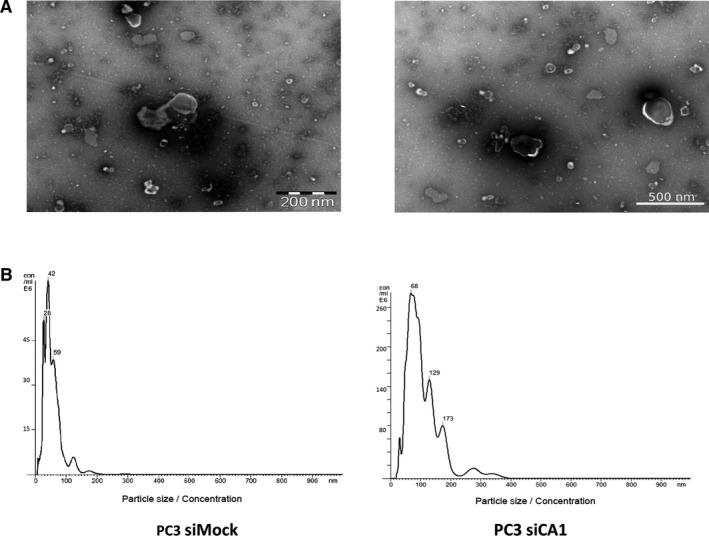
Characterization of PC3 silencing of carbonic anhydrase I (siCA1) and PC3 siMock isolated exosomes (A) Representative transmission electron microscopy (TEM) images. Five microlitres of PC3 siCA1 and PC3 siMock exosomes were placed on a carbon/formvar grid, negatively stained with 1% ammonium molybdate + 0.1% trehalose and examined with a FEI Morgagni transmission electron microscope operating at 80 kV. Scale bar: 200; 500 nm. (B) Representative nanoparticle tracking analysis (NTA) of isolated exosomes. Isolated exosomes were diluted to a suitable concentration with phosphate‐buffered saline (PBS) (1800x; siCA1 as well as control siMock), and the size distribution was analysed by NTA using a NanoSight NS500 (for each sample 3 × 60 second runs; for error bars indicating ±standard error of the mean/mode and final nanoparticle concentration see Figure 3)

**Figure 3:**
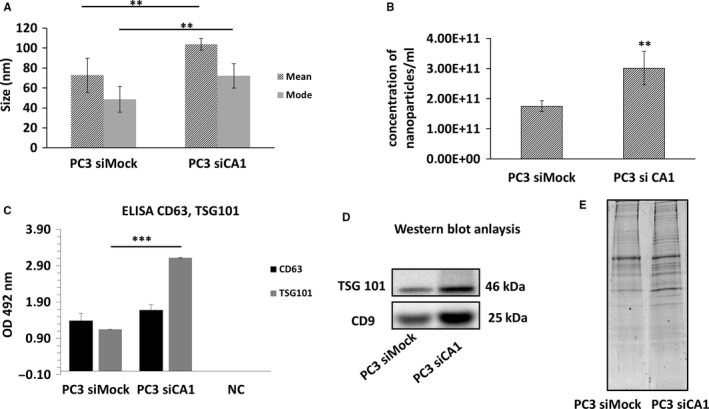
Comparative characterization of PC3 silencing of carbonic anhydrase I (siCA1) and PC3 siMock isolated exosomes. (A) Exosomal size determined by nanoparticle tracking analysis (NTA). Comparison of vesicle sizes shows that PC3 siCA1 exosomes are significantly larger than exosomes isolated from control PC3 siMock cells (unpaired *t* test, mean: PC3 siCA1 vs. PC3 siMock, *P* = 0.005200259, *P* < 0.01**; mode: PC3 siCA1 vs. PC3 siMock, *P* = 0.00914921, *P* < 0.01**). (B) The concentrations of nano‐sized particles in exosome suspension measured by NanoSight system. The concentration of PC3 si CA1 nanoparticles was significantly higher in comparison to nanoparticles from PC3 siMock control cells (PC3 siCA1 average concentration = 3.02E+11, SD = 5.48E+10; PC3 siMock average concentration = 1.75E+11, SD = 1.81E+10; unpaired *t* test, PC3 siCA1 vs. PC3 siMock, *P* = 0.006857836, *P* < 0.01**). Values are mean ± standard deviation, all values are representative of three independent experiments with three replicates. (C) Verification of exosome specific markers CD63 and TSG 101 by ELISA. The absorbance at 492 nm was measured with a xMark Microplate absorbance spectrophotometre. NC‐negative control. (D) Western Blot analysis demonstrating the expression of TSG 101 and CD 9 markers in PC3 siCA1 vs. PC3 siMock derived exosomes. (E) SDS‐PAGE analysis of exosomal protein pattern. Protein (20 µg) from exosomes prepared from PC3 siCA1 or PC3 siMock cells were separated by 12% SDS‐PAGE and stained by Coomassie Blue to illustrate the differences in protein profile

To validate the exosome isolation and purification, transmission electron microscopy (TEM) and NTA were performed. At first, the isolated exosomes derived from PC3 siCA1 and PC3 siMock cells were fixed onto formvar‐coated carbon EM grids and visualized by TEM. Electron microscopy revealed a homogenous mixture of cup‐shaped, rounded nanovesicles with diameters varying between 30 and 150 nm (Figure 2A). Subsequently, NTA was carried out using a NanoSight NS500 (3 × 60 seconds runs) (Figure 2B) which confirmed the size distribution with a mean = 104 nm, (σ = 6 nm, n = 6); average mode = 72 nm (*σ* = 12 nm) for the PC3 siCA1 exosomes (n = 6). The PC3 siMock exosomes (n = 6) showed smaller dimensions with a mean = 73nm, (*σ* = 17 nm) and average mode = 49 nm (*σ* = 13 nm). Thus, the comparison clearly demonstrated that PC3 siCA1 cells produced significantly larger exosomes than the PC3 siMock cells (unpaired *t* test, mean: PC3 siCA1 vs. PC3 siMock, *P* = 0.005, *P* < 0.01**; mode: PC3 siCA1 vs. PC3 siMock, *P* = 0.009, *P* < 0.01**) (Figure 3A).

A NanoSight system was also used for the nano‐sized particle quantity determination in the suspension of isolated exosomes. The concentration of the nano‐size particles derived from PC3 siCA1 cells was significantly higher than from PC3 siMock control cells (unpaired *t* test, PC3 siCA1 vs. PC3 siMock, *P* = 0.007, *P* < 0.01**). The average concentration was established as 3.02E+11 particles/mL (*σ* = 5.48E+10 particles/mL) and 1.75E+11 particles/mL (*σ* = 1.81E+10 particles/mL) for the suspensions isolated from the silenced and the control cells, respectively. The values are presented as means ± σ of three biological and technical replicates (Figure 3B).

Furthermore, the amount of total protein was quantified in the isolated nanovesicles by BCA assay and the presence of exosome specific markers CD9, CD63 (both tetraspanins) and TSG101 (tumour susceptibility gene 101 protein) was verified using Western Blot analyses and ELISA assay. As shown in Figure 3C,D, the level of these markers as well as the total protein content (Figure 3E) were higher in the exosomal suspension derived from CA1 siRNA transfected cells compared with control PC3 siMock cells. Significant differences were also found between protein patterns of CA1 silenced and control cells (Figure 3E) separated in 12% SDS‐PAGE.

Generally, these molecular and biophysical measurements demonstrate that the silencing of the CA1 gene in PC3 prostate cells has a profound effect on the production of exosomes and their secretion into the cultivation medium. It is also noteworthy that the method of centrifugation, filtration, concentration of the culture medium and final isolation using an ExoQuick seems to be reliable for the purification of high‐quality exosomes.

### 3.3 CA1 silencing in prostatic cancer cells alters the protein cargo of the exosomes

To identify changes in the protein composition of exosomes secreted by prostatic PC3 cells in relation to the expression status of the CA1 gene, a comparative proteomic analysis was performed. The isolated exosomes were lysed by sonication and the protein cargo digested by trypsin. Subsequent LC‐MS/MS analyses of the generated peptide mixture resulted in the identification of 196 proteins with more than two matching peptides (Table S1). Among them, 42 proteins demonstrated statistically significant (*P* < 0.05, ≥1.5‐fold change) differences (Table 1). Interestingly, almost all of them (41 proteins) were more abundant in PC3 siCA1‐derived exosomes compared with controls.

**Table 1:** Identification of exosome—associated proteins.

Accession	Peptides	Score	ANOVA (p)	Fold	log 2 (fold)	Description/molecular function	Average normalised abundances		Localization
							Mock	siCA1	
*Binding*									
P63104	5 (4)	43.73	6.32E−03	2.08	1.06	14‐3‐3 protein zeta/delta OS = Homo sapiens GN = YWHAZ PE = 1 SV = 1	532.14	1104.66	Cytoplasm
P43686	4 (4)	33.23	7.16E−03	2.16	1.11	26S protease regulatory subunit 6B OS = Homo sapiens GN = PSMC4 PE = 1 SV = 2	367.91	796.05	Nucleus
P35998	2 (2)	12.88	1.88E−03	1.99	0.99	26S protease regulatory subunit 7 OS = Homo sapiens GN = PSMC2 PE = 1 SV = 3	437.08	869.42	Cytoplasm
P07355	7 (6)	70.95	3.01E−03	2.13	1.09	Annexin A2 OS = Homo sapiens GN = ANXA2 PE = 1 SV = 2	1131.16	2409.8	Extracellular matrix, secretion
P01024	2 (2)	19.10	0.01	3.09	1.63	Complement C3 OS = Homo sapiens GN = C3 PE = 1 SV = 2	457.65	1414.35	Extracellular matrix, secretion
P62807	7 (6)	67.80	0.03	1.90	0.93	Histone H2B OS = Homo sapiens GN = HIST1H2BF PE = 2 SV = 1	2365.09	4503.56	Nucleus
P62805	5 (5)	53.81	0.04	3.31	1.73	Histone H4 OS = Homo sapiens GN = HIST1H4A PE = 1 SV = 2	578.73	174.87	Nucleus
P0DMV9	7 (3)	61.09	0.04	1.86	0.90	Heat shock 70 kDa protein 1B OS = Homo sapiens GN = HSPA1B PE = 1 SV = 1	391.3	726.97	Cytoskeletion, centrosome
P04792	5 (4)	55.78	0.03	1.85	0.89	Heat shock protein beta‐1 OS = Homo sapiens GN = HSPB1 PE = 1 SV = 2	660.19	1220.52	Nucleus, cytoplasm
P31943	2 (2)	13.70	0.03	1.93	0.95	Heterogeneous nuclear ribonucleoprotein H OS = Homo sapiens GN = HNRNPH1 PE = 1 SV = 1	161.52	312.05	Nucleus
Q14974	6 (6)	48.45	0.04	1.58	0.66	Importin subunit beta‐1 OS = Homo sapiens GN = KPNB1 PE = 1 SV = 2	618.02	979.23	Nucleus, cytoplasm
P01130	4 (4)	33.29	3.17E−03	2.74	1.45	Low‐density lipoprotein receptor OS = Homo sapiens GN = LDLR PE = 1 SV = 1	243.49	667.06	Membrane, endosomes, GA
P35579	6 (6)	45.79	1.31E−06	3.24	1.70	Myosin‐9 OS = Homo sapiens GN = MYH9 PE = 1 SV = 4	303.04	981.28	Cytoskeletion
Q15149	2 (2)	18.81	4.58E−03	2.61	1.38	Plectin (Fragment) OS = Homo sapiens GN = PLEC PE = 1 SV = 1	252.03	657.22	Cytoskeletion
P09874	2 (2)	18.84	0.01	2.84	1.51	Poly [ADP‐ribose] polymerase 1 OS = Homo sapiens GN = PARP1 PE = 1 SV = 4	105.09	298.46	Nucleus
Q16799	2 (1)	24.71	1.30E−05	2.77	1.47	Reticulon OS = Homo sapiens GN = RTN1 PE = 1 SV = 1	286.49	794.43	Endoplasmatic reticulum
P62140	2 (2)	20.67	2.14E−05	5.00	2.32	Serine/threonine‐protein phosphatase PP1‐beta catalytic subunit OS = Homo sapiens GN = PPP1CB PE = 1 SV = 3	299.16	1495.44	Nucleus
P48643	8 (6)	73.19	3.41E−03	1.61	0.69	T‐complex protein 1 subunit epsilon OS = Homo sapiens GN = CCT5 PE = 1 SV = 1	1366.32	2204.6	Cytoskeletion
Q99832	7 (6)	68.33	5.85E−03	2.29	1.20	T‐complex protein 1 subunit eta OS = Homo sapiens GN = CCT7 PE = 1 SV = 2	864.32	1975.35	Cytoplasm
P78371	7 (6)	61.93	0.04	1.50	0.58	T‐complex protein 1 subunit beta OS = Homo sapiens GN = CCT2 PE = 1 SV = 4	908.71	1366.06	Cytoplasm
Q9BUF5	9 (2)	66.65	6.31E−03	3.63	1.86	Tubulin beta‐6 chain OS = Homo sapiens GN = TUBB6 PE = 1 SV = 1	47.35	171.88	Cytoskeletion
P07996	2 (2)	13.57	1.27E−04	2.53	1.34	Thrombospondin‐1 OS = Homo sapiens GN = THBS1 PE = 1 SV = 2	298.54	755.51	Endoplasmatic reticulum extracellular matrix
P13010	11 (11)	99.67	4.46E−03	1.95	0.96	X‐ray repair cross‐complementing protein 5 OS = Homo sapiens GN = XRCC5 PE = 1 SV = 3	1029.18	2002.42	Nucleus
Q9Y2P7	2 (1)	18.82	1.41E−04	3.75	1.91	Zinc finger protein 256 OS = Homo sapiens GN = ZNF256 PE = 4 SV = 1	19.27	72.33	Nucleus
*Catalytic activity*									
P43686	4 (4)	33.23	7.16E−03	2.16	1.11	26S protease regulatory subunit 6B OS = Homo sapiens GN = PSMC4 PE = 1 SV = 2	367.91	796.05	Nucleus
P35998	2 (2)	12.88	1.88E−03	1.99	0.99	26S protease regulatory subunit 7 OS = Homo sapiens GN = PSMC2 PE = 1 SV = 3	437.08	869.42	Cytoplasm
P01024	2 (2)	19.10	0.01	3.09	1.63	Complement C3 OS = Homo sapiens GN = C3 PE = 1 SV = 2	457.65	1414.35	Extracellular matrix, secretion
P11586	2 (2)	18.33	3.43E−03	2.41	1.27	C‐1‐tetrahydrofolate synthase_ cytoplasmic OS = Homo sapiens GN = MTHFD1 PE = 1 SV = 1	119.51	288.08	Cytoplasm
P26641	7 (6)	58.66	1.56E−04	1.98	0.99	Elongation factor 1‐gamma OS = Homo sapiens GN = EEF1G PE = 1 SV = 3	559.55	1108.5	Cytoplasm
P0DMV9	7 (3)	61.09	0.04	1.86	0.90	Heat shock 70 kDa protein 1B OS = Homo sapiens GN = HSPA1B PE = 1 SV = 1	391.3	726.97	Cytoskeletion, centrosome
P04792	5 (4)	55.78	0.03	1.85	0.89	Heat shock protein beta‐1 OS = Homo sapiens GN = HSPB1 PE = 1 SV = 2	660.19	1220.52	Nucleus, cytoplasm
P35579	6 (6)	45.79	1.31E−06	3.24	1.70	Myosin‐9 OS = Homo sapiens GN = MYH9 PE = 1 SV = 4	303.04	981.28	Cytoskeletion
Q14697	4 (4)	34.09	0.01	2.57	1.36	Neutral alpha‐glucosidase AB OS = Homo sapiens GN = GANAB PE = 1 SV = 1	194.31	499.1	Endoplasmatic reticulum, Golgi aparatus
P14618	22 (20)	224.48	2.91E−06	1.99	0.99	Pyruvate kinase PKM OS = Homo sapiens GN = PKM PE = 1 SV = 4	3161.71	6281.27	Cytoplasm
Q92626	12 (12)	101.96	2.13E−04	2.07	1.05	Peroxidasin homolog OS = Homo sapiens GN = PXDN PE = 1 SV = 2	1346.04	2780.28	Extracelular matrix, secretion
O14818	7 (6)	71.05	4.55E−03	2.46	1.30	Proteasome subunit alpha type‐7 OS = Homo sapiens GN = PSMA7 PE = 1 SV = 1	887.97	2180.2	Nucleus
P25786	5 (5)	54.34	2.06E−03	1.97	0.98	Proteasome subunit alpha type‐1 OS = Homo sapiens GN = PSMA1 PE = 1 SV = 1	1220.78	2401.13	Nucleus
P21980	3 (3)	19.12	5.80E−06	3.60	1.85	Protein‐glutamine gamma‐glutamyltransferase 2 OS = Homo sapiens GN = TGM2 PE = 1 SV = 2	194.67	700.1	Mitochondrion, cytoplasm
P62140	2 (2)	20.67	2.14E−05	5.00	2.32	Serine/threonine‐protein phosphatase PP1‐beta catalytic subunit OS = Homo sapiens GN = PPP1CB PE = 1 SV = 3	299.16	1495.44	Nucleus
P26639	2 (2)	20.76	1.93E−03	2.96	1.57	Threonine—tRNA ligase cytoplasmic OS = Homo sapiens GN = TARS PE = 1 SV = 3	263.88	781.32	Cytoplasm
P60174	2 (2)	16.70	0.03	14.94	3.90	Triosephosphate isomerase OS = Homo sapiens GN = TPI1 PE = 1 SV = 3	24.27	362.52	Cytoplasm
*Structural molecule activity*									
P08865	9 (8)	78.96	2.50E−03	1.91	0.93	40S ribosomal protein SA OS = Homo sapiens GN = RPSA PE = 1 SV = 1	1045.46	1995.95	Nucleus
P68032	11 (4)	110.00	5.36E−03	2.78	1.48	Actin_ alpha cardiac muscle 1 OS = Homo sapiens GN = ACTC1 PE = 1 SV = 1	584.62	1626.59	Cytoskeletion
P31943	2 (2)	13.70	0.03	1.93	0.95	Heterogeneous nuclear ribonucleoprotein H OS = Homo sapiens GN = HNRNPH1 PE = 1 SV = 1	161.52	312.05	Nucleus
Q15149	2 (2)	18.81	4.58E−03	2.61	1.38	Plectin (Fragment) OS = Homo sapiens GN = PLEC PE = 1 SV = 1	252.03	657.22	Cytoskeletion
Q9BUF5	9 (2)	66.65	6.31E−03	3.63	1.86	Tubulin beta‐6 chain OS = Homo sapiens GN = TUBB6 PE = 1 SV = 1	47.35	171.88	Cytoskeletion
*Translation regulator activity*									
P13639	18 (17)	191.62	5.30E−03	2.74	1.45	Elongation factor 2 OS = Homo sapiens GN = EEF2 PE = 1 SV = 4	3820.7	1.05E+04	Nucleus
*Protein‐protein interaction*									
Q6S8J3	13 (3)	138.63	1.01E−06	2.91	1.54	POTE ankyrin domain family member E OS = Homo sapiens GN = POTEE PE = 1 SV = 3	142.49	414.62	Extracellular matrix, secretion
*Response to stress*									
P14625	13 (12)	129.22	0.02	1.72	0.78	Endoplasmin OS = Homo sapiens GN = HSP90B1 PE = 1 SV = 1	1779.22	3061.84	Endoplasmatic reticulum
*Antioxidant*									
Q92626	12 (12)	101.96	2.13E−04	2.07	1.05	Peroxidasin homolog OS = Homo sapiens GN = PXDN PE = 1 SV = 2	1346.04	2780.28	Extracelular matrix, secretion
*Transporter activity*									
Q14974	6 (6)	48.45	0.04	1.58	0.66	Importin subunit beta‐1 OS = Homo sapiens GN = KPNB1 PE = 1 SV = 2	618.02	979.23	Nucleus, cytoplasm
*Extracelular matrix structural costituent*									
P24821	5 (4)	46.13	2.93E−03	3.4	1.77	Tenascin OS = Homo sapiens GN = TNC PE = 1 SV = 3	190.92	649.52	Extracellular matrix, secretion
*Receptor activity*									
P01130	4 (4)	33.29	3.17E−03	2.74	1.45	Low‐density lipoprotein receptor OS = Homo sapiens GN = LDLR PE = 1 SV = 1	243.49	667.06	Membrane, endosomes, GA

Proteomic profiles from PC3 siCA1 and control PC3 siMock derived exosomes were compared. Proteins with more than 2 matching peptides, statistically significant (p < 0.05) and with more than 1.5‐fold expression (and log2 transformed fold change), were further functionally categorized and listed in this table.

**Figure 4:**
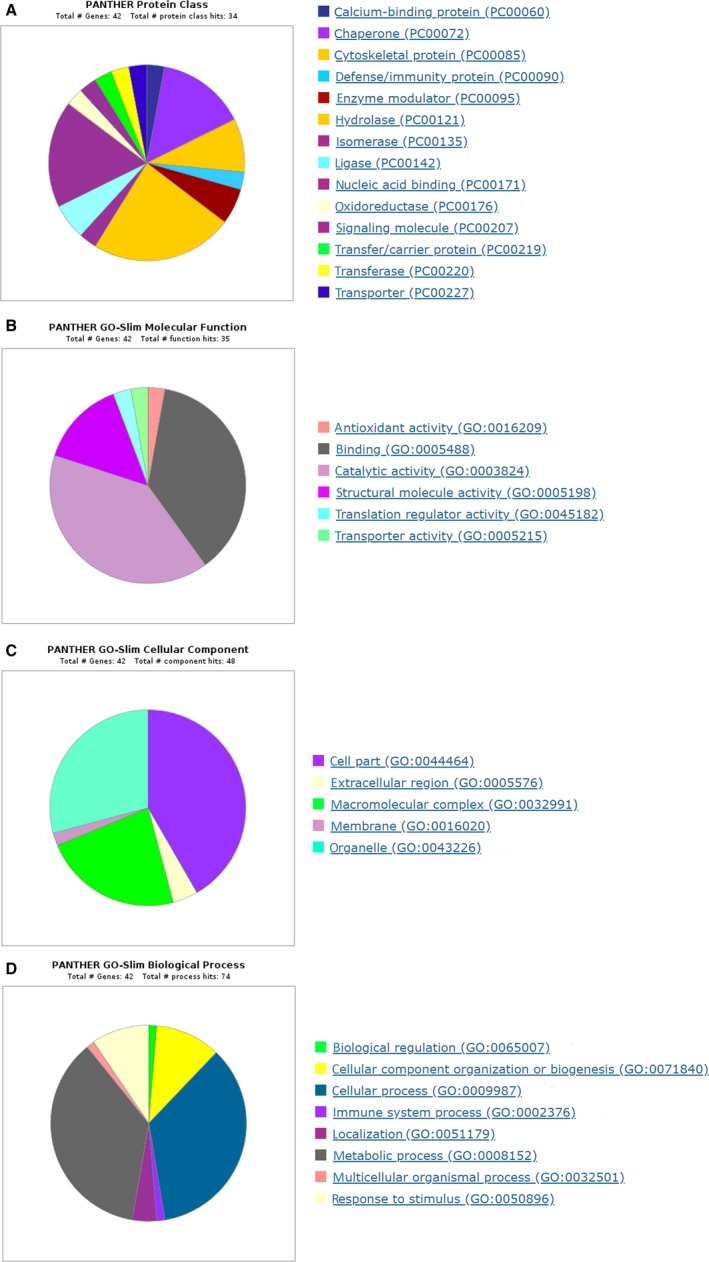
PANTHER gene ontology enrichment analysis of statistically changed proteins from PC3 silencing of carbonic anhydrase I (siCA1) derived exosomes. Enrichment analyses were performed in terms of protein class (A), molecular function (B), cellular component (C) and biological process (D)

A gene ontology (GO) database search was carried out to reveal molecular functions and cellular localization of the identified proteins as well as the biological processes in which they are involved. Here it is important to mention that GO annotations often provide various cellular locations and molecular functions for a particular protein. The largest proportion of the identified proteins are enzymes (hydrolases (elongation factor 2, 26S protease regulatory subunit 7, proteasome subunit alpha type‐1, serine/threonine‐protein phosphatase PP1‐beta catalytic subunit, proteasome subunit alpha type‐7, neutral alpha‐glucosidase AB, 26S protease regulatory subunit 6B, proteasome subunit beta type‐2), an isomerase (triosephosphate isomerase [TPI]), ligases (C‐1‐tetrahydrofolate synthase/cytoplasmic, threonine‐tRNA ligase/cytoplasmic), transferases/oxidoreductases (protein‐glutamine gamma‐glutamyltransferase 2 [TGM2], peroxidasin homolog); 38.1%), nucleic acid binding proteins (elongation factor 2, histone H3.2, histone H4, histone H2B type 1‐C/E/F/G/I, X‐ray repair cross‐complementing protein 5, heterogeneous nuclear ribonucleoprotein H; 17.6%) and chaperones (endoplasmin, T‐complex protein 1 subunit beta, 14‐3‐3 protein zeta/delta, T‐complex protein 1 subunit eta, T‐complex protein 1 subunit epsilon; 14.7%). Proteins involved in cytoskeleton structure (actin, alpha cardiac muscle 1, tubulin, beta‐6 chain, plectin; 8.8%), modulation of enzymes (elongation factor 2, complement C3; 5.9%), or defense/immunity (complement C3; 2.9%) comprise the second most represented group. The last class was made up of a transfer/carrier protein (importin subunit beta‐1; 2.9%), a calcium‐binding protein (serine/threonine‐protein phosphatase PP1‐beta catalytic subunit; 2.9%), a transporter (importin subunit beta‐1; 2.9%) and a signal molecule (complement C3; 2.9%) (Figure 4A). Therefore, most of the identified proteins have catalytic, binding or structural functions, while the antioxidant, translation and transporter regulatory activities are less frequent (Figure 4B). Regarding the cellular location (Figure 4C), the majority of the identified proteins are predicted to be located in the cytoplasm (41.7%) or organelles (29.7%). Further bioinformatics characterization assigned these proteins to the following biological process: cellular processes (35.1%), metabolic processes (36.5%), cellular component organization or biogenesis (10.8%) and response to stimuli (9.5%) (Figure 4D).

## 4 DISCUSSION

We have previously shown that the presence of autoantibodies against CA I can be correlated with the stage of the malignant disease. It was noticed, that high titers of these autoantibodies in the sera of patients can be associated with a good prognosis which is usually accompanied with spontaneous regression of the tumour after high dose therapy and autologous stem cell transplantation.^21^ To shed more light onto this phenomenon, we have treated several cancer cell lines derived from prostatic, colon or breast carcinomas with the sera of these patients. Significant modifications in the morphology of these cells were noticed in contrast to controls. The mRNA levels for proteins associated with basal lamina assembly, cytoskeleton, WNT7B and CTHRC1 were down‐regulated, whereas the expression of CA1 mRNA was up‐regulated in the tumour cell lines treated with the anti‐CA I autoantibody positive sera.^22^ To examine the effect of the opposite phenomenon, the CA1 mRNA of PC3 prostatic cancer cells was directly targeted and silenced using the RNA interference system. The results show that the knock‐down of the CA1 gene enhances or in some cases only slightly changes the gene expression of the ECM proteins.^23^

Exosomes produced by tumours carry cargoes that partially mimic the contents of parent cells and act as messengers in both homeostasis and pathophysiological conditions. Because these exosomes are found in all body fluids, they are of potential interest as non‐invasive biomarkers of the cancer cell status.^27^ In this study, we compared the exosomes originating from PC3 cells with silenced CA1 mRNA and from the PC3 siMock cells to assess the impact of reduced CA I expression. Using a NanoSight system, we observed altered sizes and concentrations of particles in exosomes from PC3 cells with silenced CA1 mRNA. In this comparison, PC3 siCA1 exosomes were significantly larger than exosomes isolated from control PC3 siMock cells (Figure 2). Similarly, the concentration of nanoparticles derived from PC3 siCA1 cells was also significantly higher compared with controls. Further analyses using ELISA and WB showed that the markers CD9, CD63, TSG 101 are expressed more in the exosomes derived from CA1 siRNA transfected cells (Figure 3). Thus these findings confirm that the reduction in CA1 gene expression has a significant effect on the formation of exosomes and their secretion into the environment.

As mentioned above, the LC‐MS/MS analyses of the exosomal cargo identified 196 proteins. This result is in accordance with the published datasets (www.exocarta.org) on exosomes of prostatic cancer cell lines/sera/urine. It is noteworthy that almost all significantly altered proteins due to CA I reduction (Table 1) are known cancer markers or have been associated with prostate or other malignant diseases.

For instance, the eukaryotic elongation factor 2 (eEF2), a key regulator of protein synthesis, was correlated with the progression of several types of cancer. Recently, it has been reported that the eEF2 protein is highly expressed in human breast, lung, gastric and colorectal carcinoma tissues, but not in normal tissues. For this reason, it has been suggested as an effective target for immunotherapy.^28^ Additionally, Zhang et al^29^ demonstrated the expression of the eEF2 protein in prostatic carcinoma tissue by immunohistochemistry. The authors correlated this expression with clinicopathological parameters of prostatic cancer patients and proposed this protein as a potential biomarker of prostate cancer. Moreover, a proteasome subunit alpha was found to be significantly altered due to reduced CA I expression in this study. This protein is a constituent of the ubiquitin‐proteasome pathway which is essential for cell growth, cell viability and many other biological processes.^30^ Deregulation or dysfunction in this pathway may result in severe pathological conditions, such as immune defects, neurodegenerative disorders and cancer.^31^, ^32^ Recent data show a close relationship between the enhanced proteasome activity and overexpression of proteasome subunit proteins. For example, the elevated expression level of certain proteasome subunits was found in the samples of patients with breast cancer along with higher proteasome activity.^32^ The overexpression of proteasome subunit S10 accompanied by an increased proteasome activity has also been reported in melanoma.^33^ It is noteworthy, that the application of a proteasome inhibitor suppresses the transactivation of the androgen receptor (AR) in an androgen‐dependent manner in prostate cancer LNCaP and PC3 cell lines. Thus, the proteasome system seems to play an important role in the regulation of AR activity and can be proposed as a unique target for the development of therapeutic drugs blocking androgen/AR‐mediated prostate tumour growth.^34^ Because translation is the final step in the production of a functional protein, alterations in translational control may represent an ‘oncogenic' node which may serve as a potential target for tumour suppression. Ultimately, the target of specific translational components in cancer represents the most promising therapeutic approaches for clinical trials.^35^

In PC3 siCA1 derived exosomes we also found an increased level of the poly [ADP‐ribose] polymerase 1 (PARP‐1). This nuclear enzyme is involved in transcription regulation and DNA repair. Lavery et al^36^ have reported that 15% to 20% of metastatic prostate cancers are characterized by a deficiency in DNA repair genes, which make them susceptible to DNA‐damaging therapies. It was found that PARP 1 expression is significantly increased in several malignant tissues, including prostate, breast, uterine, lung, ovarian and skin cancers and non‐Hodgkin's lymphoma.^37^ Recent studies also point out that this enzyme may contribute to oncogenic signalling and cancer progression. On the other hand, the activity of PARP‐1 can be deregulated by PARP inhibitors which act through a synthetic lethal mechanism of action. This leads to the inability of tumour cells with specific genetic mutations to repair DNA double‐strand breaks in various cancers, making these inhibitions a suitable target for novel cancer therapies.^38^, ^39^

Tumour cells undergo different genetic and metabolic alterations that directly contribute to their growth and malignancy. Metabolic reprogramming accompanied by a shift from oxidative phosphorylation towards aerobic glycolysis, mutations in the tricarboxylic acid cycle metabolic enzymes and addiction from lipid and glutamine metabolism are the key characteristics of cancer cells. In this study, we identified several metabolic enzymes with higher abundance due to knockdown of CA1 mRNA that may play an important role in tumour progression: TPI, protein‐glutamine‐gamma‐glutamyltransferase 2, peroxidasin homolog and pyruvate kinase (PKM). PKM is essential for aerobic glycolysis, a dominant metabolic pathway utilized by cancer cells. Besides this role, PKM has moonlighting functions, including gene transcription that may promote cancer.^40^ Indeed, using immunohistochemical staining, Wong et al^41^ detected higher levels of PKM in aggressive xenograft tumours derived from prostatic cancer stem‐like cells (PCSCs) compared to non‐PCSCs. Thus, this finding suggests that up‐regulation and specific modification of PKM may result in prostatic cancer progression. Furthermore, several studies have demonstrated that TPI is overexpressed in many cancers, such as lung adenocarcinoma, bladder squamous cell carcinoma and breast carcinoma. Increased abundance of TPI is in line with tumour development associated with tumour cell proliferation, migration and invasion.^42^, ^43^ TGM2 is also an active player in boosting chemo‐resistance or malignant cell mobility and invasion mainly through induction in epithelial‐mesenchymal transition (EMT). Aberrant regulation of TGM2 gene has been documented in various cancer types, particularly those isolated from metastatic or chemo‐resistance sites.^44^ Peroxidasin (PXDN), as an ECM‐linked peroxidase, plays an important role in several biological processes including apoptosis and consolidation of the ECM.^45^ Dysregulation and/or mutations of PXDN have been accompanied with various pathologies that promote EMT, fibrosis or cancer (brain, melanoma, acute myeloid leukemia).^46^, ^47^ Knockdown of PXDN expression may lead to cell detachment in choriocarcinoma, while reduced PXDN in melanoma was correlated with decreased cell invasion.^46^, ^47^, ^50^

Molecular chaperones and heat shock proteins (HSP) represented by endoplasmin, heat shock 70 kDa protein 1B, heat shock protein beta‐1, T‐complex protein 1 subunit beta, T‐complex protein 1 subunit eta and T‐complex protein 1 subunit epsilon were also identified in this study as differentially altered due to reduced levels of CA I. HSPs are known to be expressed at the high amount in a wide range of tumours. They are in close association with a poor prognosis and resistance to therapy. The HSP family members participate in autonomous cell proliferation and inhibit death pathways.^51^ Also, 14‐3‐3 protein zeta/delta which was more abundant in PC3 siCA1 derived exosomes promotes cell proliferation, adhesion and survival and inhibits apoptosis in multiple cancers. For this function represents a novel molecular agent for targeted cancer therapy.^52^

In addition to the above‐mentioned proteins, we observed an increase in the abundance of nucleic acid binding proteins (histone H3.2, histone H2B type 1‐C/E/F/G/I, X‐ray repair cross‐complementing protein 5, heterogeneous nuclear ribonucleoprotein H). These are key players in chromatin structure shaping. They regulate fundamental cellular processes such as chromosome segregation and gene expression. For these reasons, histone variants represent potential drivers of cancer initiation and/or progression. Thus, targeting histone deposition or the chromatin remodelling machinery may have a therapeutic value.^53^

Lastly, among the differentially abundant proteins, are the constituents of the cell cytoskeleton (actin, alpha cardiac muscle 1, tubulin, beta‐6 chain, plectin), defense/immunity protein (complement C3) and transfer/carrier (importin subunit beta‐1). Mutations and the abnormal expression of cytoskeletal and cytoskeletal‐associated proteins play an important role in the ability of cancer cells to resist chemotherapy and metastasize. The dynamic reorganization of the actin cytoskeleton is a prerequisite for the morphology, migration and invasion of cancer cells.^54^ The complement 3 (C3), as a central protein of the complement system, is expressed in numerous cancer tissues (lung, colorectal, esophageal and gastric) indicating that C3 may be a suitable biomarker for the outcome of malignancies.^55^ Moreover, up‐regulation of importin subunit beta‐1 promotes tumour cell proliferation and predicts poor prognosis in non‐small lung, cervical, glioma and gastric cancer.^56^, ^57^ All these proteins are known as building blocks for cell formation and survival.

## 5 CONCLUSION

A comprehensive characterization of the exosomes derived from a PC3 prostate cancer cell line demonstrated that the knockdown of CA1 mRNA affects the size and concentration of exosomes as well as the protein repertoire in their cargo. Notably, almost all differentially abundant proteins have been described as ‘enhancers' of tumour development due to their promotion of tumour cell proliferation, migration and invasion. Based on these results we propose that the reduced expression of the CA I protein in PC3 cells through various cascades enhances the malignant potential of prostatic cancer cells. Although the exact mechanism remains unclear, we believe that CA1 mRNA and/or the enzyme CA I play a crucial role in the malignancy of PC3 prostatic cells and might present a novel strategy for the future treatment of prostatic cancer.

## CONFLICT OF INTEREST

The authors confirm that there are no conflicts of interest.
